# Supplementary material for: The variability of emotions, physical complaints, intention, and self-efficacy: an ecological momentary assessment study in older adults
Source: PeerJ. 2022 May 19;10:e13234. doi: 10.7717/peerj.13234 (PMC9124457; doi:10.7717/peerj.13234)
Supplement: Supplemental Information 2 [file peerj-10-13234-s002.docx]

1. Paper-based questionnaire
2. What is your gender?

- Male
- Female

1. How old are you? _____________ years
2. How tall are you? ____________ cm
3. How much do you weigh? ____________ kg
4. What was your main occupation before retirement?

- Employee
- Self-employed
- Education
- Worker
- Executives
- Free profession
- Officer
- Household
- Unemloyed
- Disabled
- Other: ______________________________________

1. What is the highest diploma or certificate you have obtained?

- None
- Primary education
- Secundairy education
- Higher education
- University education
- Other: ______________________________________

1. What is your marital status?

- Single
- Married or living together
- Divorced
- Widow/widower

1. How many children do you have? ____________ children
2. How many grandchildren do you have? ____________ grandchildren
3. Do you have pets?

- Yes
- No

🡪 If yes, which ones? ________________________

1. EMA questionnaire
2. How **cheerful** were you just before you received the trigger?

| Not cheerful at all |  | A little cheerful |  | Quite cheerful |  | Very cheerful |
| --- | --- | --- | --- | --- | --- | --- |
| 1 | 2 | 3 | 4 | 5 | 6 | 7 |

1. How **relaxed** were you just before you received the trigger?

| Not relaxed at all |  | A little relaxed |  | Quite relaxed |  | Very relaxed |
| --- | --- | --- | --- | --- | --- | --- |
| 1 | 2 | 3 | 4 | 5 | 6 | 7 |

1. How **enthusiastic** were you just before you received the trigger?

| Not enthusiastic at all |  | A little enthusiastic |  | Quite enthusiastic |  | Very enthusiastic |
| --- | --- | --- | --- | --- | --- | --- |
| 1 | 2 | 3 | 4 | 5 | 6 | 7 |

1. How **satisfied** were you just before you received the trigger?

| Not satisfied at all |  | A little satisfied |  | Quite satisfied |  | Very satisfied |
| --- | --- | --- | --- | --- | --- | --- |
| 1 | 2 | 3 | 4 | 5 | 6 | 7 |

1. How **insecure** were you just before you received the trigger?

| Not insecure at all |  | A little insecure |  | Quite insecure |  | Very insecure |
| --- | --- | --- | --- | --- | --- | --- |
| 1 | 2 | 3 | 4 | 5 | 6 | 7 |

1. How **anxious** were you just before you received the trigger?

| Not anxious at all |  | A little anxious |  | Quite anxious |  | Very anxious |
| --- | --- | --- | --- | --- | --- | --- |
| 1 | 2 | 3 | 4 | 5 | 6 | 7 |

1. How **irritated** were you just before you received the trigger?

| Not irritated at all |  | A little irritated |  | Quite irritated |  | Very irritated |
| --- | --- | --- | --- | --- | --- | --- |
| 1 | 2 | 3 | 4 | 5 | 6 | 7 |

1. How **down** were you **feeling** just before you received the trigger?

| Not feeling down at all |  | Feeling a little down |  | Feeling quite down |  | Feeling very down |
| --- | --- | --- | --- | --- | --- | --- |
| 1 | 2 | 3 | 4 | 5 | 6 | 7 |

1. How **fatigued** did you feel just before you received the trigger?

| Not fatigued at all |  | A little fatigued |  | Quite fatigued |  | Very fatigued |
| --- | --- | --- | --- | --- | --- | --- |
| 1 | 2 | 3 | 4 | 5 | 6 | 7 |

1. How much **pain** did you have just before you received the trigger?

| No pain |  | A little pain |  | Quite some pain |  | A lot of pain |
| --- | --- | --- | --- | --- | --- | --- |
| 1 | 2 | 3 | 4 | 5 | 6 | 7 |

1. How **dizzy** were you feeling just before you received the trigger?

| Not dizzy at all |  | A little dizzy |  | Quite dizzy |  | Very dizzy |
| --- | --- | --- | --- | --- | --- | --- |
| 1 | 2 | 3 | 4 | 5 | 6 | 7 |

1. How **stiff** were you just before you received the trigger?

| Not stiff at all |  | A little stiff |  | Quite stiff |  | Very stiff |
| --- | --- | --- | --- | --- | --- | --- |
| 1 | 2 | 3 | 4 | 5 | 6 | 7 |

1. How **short of breath** were you just before you received the trigger?

| Not short of breath at all |  | A little short of breath |  | Quite short of breath |  | Very short of breath |
| --- | --- | --- | --- | --- | --- | --- |
| 1 | 2 | 3 | 4 | 5 | 6 | 7 |

1. In the next two hours, I **can** move for at least 10 minutes.

| Strongly disagree |  | A little agree |  | Agree |  | Strongly agree |
| --- | --- | --- | --- | --- | --- | --- |
| 1 | 2 | 3 | 4 | 5 | 6 | 7 |

1. In the next two hours, I **will** move for at least 10 minutes

| Strongly disagree |  | A little agree |  | Agree |  | Strongly agree |
| --- | --- | --- | --- | --- | --- | --- |
| 1 | 2 | 3 | 4 | 5 | 6 | 7 |
