# Supplementary material for: The variability of emotions, physical complaints, intention, and self-efficacy: an ecological momentary assessment study in older adults
Source: PeerJ. 2022 May 19;10:e13234. doi: 10.7717/peerj.13234 (PMC9124457; doi:10.7717/peerj.13234)
Supplement: Supplemental Information 3 [file peerj-10-13234-s003.docx]

1. Paper-based questionnaire in Dutch (original language)
2. Wat is uw geslacht?

- Man
- Vrouw

1. Hoe oud bent u? _____________ jaar
2. Hoe groot bent u? ____________ cm
3. Hoeveel weegt u? ____________ kg
4. Wat was uw hoofdberoep voor uw pensioen?

- Bediende
- Zelfstandige
- Onderwijs
- Arbeider
- Kaderpersoneel
- Vrij beroep
- Ambtenaar
- Huisman/vrouw
- Werkloos
- Invalide
- Andere: ______________________________________

1. Wat is het hoogste diploma of getuigschrift dat u behaald heeft?

- Geen
- Lager onderwijs
- Secundair onderwijs
- Hoger onderwijs
- Universitair onderwijs
- Andere: ______________________________________

1. Wat is uw burgerlijke staat?

- Alleenstaand
- Gehuwd of samenwonend
- Echtgescheiden
- Weduwe/weduwnaar

1. Hoeveel kinderen heeft u? ____________ kinderen
2. Hoeveel kleinkinderen heeft u? ____________ kleinkinderen
3. Heeft u huisdieren?

- Ja
- Neen

🡪 Indien ja, welke ? ________________________

1. EMA questionnaire in Dutch (original language)
2. Hoe **opgewekt** was u juist voor de melding?

| Helemaal niet opgewekt |  | Een beetje opgewekt |  | Vrij opgewekt |  | Heel erg opgewekt |
| --- | --- | --- | --- | --- | --- | --- |
| 1 | 2 | 3 | 4 | 5 | 6 | 7 |

1. Hoe **ontspannen** was u juist voor de melding?

| Helemaal niet ontspannen |  | Een beetje ontspannen |  | Vrij ontspannen |  | Heel erg ontspannen |
| --- | --- | --- | --- | --- | --- | --- |
| 1 | 2 | 3 | 4 | 5 | 6 | 7 |

1. Hoe **enthousiast** was u juist voor de melding?

| Helemaal niet enthousiast |  | Een beetje enthousiast |  | Vrij enthousiast |  | Heel erg enthousiast |
| --- | --- | --- | --- | --- | --- | --- |
| 1 | 2 | 3 | 4 | 5 | 6 | 7 |

1. Hoe **tevreden** was u juist voor de melding?

| Helemaal niet tevreden |  | Een beetje tevreden |  | Vrij tevreden |  | Heel erg tevreden |
| --- | --- | --- | --- | --- | --- | --- |
| 1 | 2 | 3 | 4 | 5 | 6 | 7 |

1. Hoe **onzeker** was u juist voor de melding?

| Helemaal niet onzeker |  | Een beetje onzeker |  | Vrij onzeker |  | Heel erg onzeker |
| --- | --- | --- | --- | --- | --- | --- |
| 1 | 2 | 3 | 4 | 5 | 6 | 7 |

1. Hoe **angstig** was u juist voor de melding?

| Helemaal niet angstig |  | Een beetje angstig |  | Vrij angstig |  | Heel erg angstig |
| --- | --- | --- | --- | --- | --- | --- |
| 1 | 2 | 3 | 4 | 5 | 6 | 7 |

1. Hoe **geïrriteerd** was u juist voor de melding?

| Helemaal niet geïrriteerd |  | Een beetje geïrriteerd |  | Vrij geïrriteerd |  | Heel erg geïrriteerd |
| --- | --- | --- | --- | --- | --- | --- |
| 1 | 2 | 3 | 4 | 5 | 6 | 7 |

1. Hoe **somber** was u juist voor de melding?

| Helemaal niet somber |  | Een beetje somber |  | Vrij somber |  | Heel erg somber |
| --- | --- | --- | --- | --- | --- | --- |
| 1 | 2 | 3 | 4 | 5 | 6 | 7 |

1. Hoe **moe** was u juist voor de melding?

| Helemaal niet moe |  | Een beetje moe |  | Vrij moe |  | Heel erg moe |
| --- | --- | --- | --- | --- | --- | --- |
| 1 | 2 | 3 | 4 | 5 | 6 | 7 |

1. Hoeveel **pijn** had u juist voor de melding?

| Helemaal geen pijn |  | Een beetje pijn |  | Veel pijn |  | Heel veel pijn |
| --- | --- | --- | --- | --- | --- | --- |
| 1 | 2 | 3 | 4 | 5 | 6 | 7 |

1. Hoe **duizelig** was u juist voor de melding?

| Helemaal niet duizelig |  | Een beetje duizelig |  | Vrij duizelig |  | Heel erg duizelig |
| --- | --- | --- | --- | --- | --- | --- |
| 1 | 2 | 3 | 4 | 5 | 6 | 7 |

1. Hoe **stijf** (spieren, gewrichten) was u juist voor de melding?

| Helemaal niet stijf |  | Een beetje stijf |  | Vrij stijf |  | Heel erg stijf |
| --- | --- | --- | --- | --- | --- | --- |
| 1 | 2 | 3 | 4 | 5 | 6 | 7 |

1. Hoe **kortademig** was u juist voor de melding?

| Helemaal niet kortademig |  | Een beetje kortademig |  | Vrij kortademig |  | Heel erg kortademig |
| --- | --- | --- | --- | --- | --- | --- |
| 1 | 2 | 3 | 4 | 5 | 6 | 7 |

1. In de komende twee uur **kan** ik minstens 10 minuten bewegen.

| Helemaal niet mee eens |  | Een beetje mee eens |  | Veel mee eens |  | Heel veel mee eens |
| --- | --- | --- | --- | --- | --- | --- |
| 1 | 2 | 3 | 4 | 5 | 6 | 7 |

1. In de komende twee uur **wil** ik minstens 10 minuten bewegen.

| Helemaal niet mee eens |  | Een beetje mee eens |  | Veel mee eens |  | Heel veel mee eens |
| --- | --- | --- | --- | --- | --- | --- |
| 1 | 2 | 3 | 4 | 5 | 6 | 7 |
