# Supplementary material for: The variability of emotions, physical complaints, intention, and self-efficacy: an ecological momentary assessment study in older adults
Source: PeerJ. 2022 May 19;10:e13234. doi: 10.7717/peerj.13234 (PMC9124457; doi:10.7717/peerj.13234)
Supplement: Supplemental Information 4 [file peerj-10-13234-s004.docx]

|  | **Participants who gave the same answer for more than 80% of the triggers** | |  | **Participants who gave the same answer for more than 80% of the triggers** | |
| --- | --- | --- | --- | --- | --- |
| **Emotions** | **N** | **%** | **Physical complaints** | **N** | **%** |
| *Cheerfulness* | 13 | 20.31 | *Fatigue* | 21 | 32.81 |
| *Relaxation* | 10 | 15.63 | *Pain* | 32 | 50.00 |
| *Enthusiasm* | 13 | 20.31 | *Dizziness* | 58 | 90.63 |
| *Satisfaction* | 9 | 14.06 | *Stiffness* | 26 | 40.63 |
| *Insecurity* | 38 | 59.38 | *Shortness of breath* | 54 | 84.38 |
| *Anxiousness* | 58 | 90.63 | **Intention and self-efficacy** | **N** | **%** |
| *Irritation* | 40 | 62.50 | *Intention* | 4 | 6.25 |
| *Feeling down* | 48 | 75 | *Self-efficacy* | 10 | 15.63 |
