# Supplementary material for: The variability of emotions, physical complaints, intention, and self-efficacy: an ecological momentary assessment study in older adults
Source: PeerJ. 2022 May 19;10:e13234. doi: 10.7717/peerj.13234 (PMC9124457; doi:10.7717/peerj.13234)
Supplement: Supplemental Information 5 [file peerj-10-13234-s005.docx]

|  | **Participants who gave the same answer for more than 70% of the triggers** | |  | **Participants who gave the same answer for more than 70% of the triggers** | |
| --- | --- | --- | --- | --- | --- |
| **Emotions** | **N** | **%** | **Physical complaints** | **N** | **%** |
| *Cheerfulness* | 18 | 28.13 | *Fatigue* | 25 | 39.06 |
| *Relaxation* | 17 | 26.56 | *Pain* | 39 | 60.94 |
| *Enthusiasm* | 18 | 28.13 | *Dizziness* | 54 | 84.38 |
| *Satisfaction* | 15 | 23.44 | *Stiffness* | 39 | 60.94 |
| *Insecurity* | 49 | 76.56 | *Shortness of breath* | 56 | 87.50 |
| *Anxiousness* | 60 | 93.75 | **Intention and self-efficacy** | **N** | **%** |
| *Irritation* | 44 | 68.75 | *Intention* | 8 | 12.50 |
| *Feeling down* | 56 | 87.50 | *Self-efficacy* | 12 | 18.75 |
