# Supplementary material for: The variability of emotions, physical complaints, intention, and self-efficacy: an ecological momentary assessment study in older adults
Source: PeerJ. 2022 May 19;10:e13234. doi: 10.7717/peerj.13234 (PMC9124457; doi:10.7717/peerj.13234)
Supplement: Supplemental Information 6 [file peerj-10-13234-s006.docx]

| **Participant** | **Number of fully completed questionnaires (freq)** | **Percentage of fully completed questionnaires (%)** | **Number of partly completed questionnaires (freq)** | **Percentage of partly completed questionnaires (freq)** |
| --- | --- | --- | --- | --- |
| 1 | 19 / 42 | 45.24 | 20 / 42 | 47.62 |
| 2 | 31 / 42 | 73.81 | 36 / 42 | 85.71 |
| 3 | 26 / 42 | 61.90 | 26 / 42 | 61.90 |
| 4 | 19 / 42 | 45.24 | 22 / 42 | 52.38 |
| 5 | 26 / 42 | 61.90 | 26 / 42 | 61.90 |
| 6 | 35 / 42 | 83.33 | 35 /42 | 83.33 |
| 7 | 34 / 42 | 80.95 | 34 / 42 | 80.95 |
| 8 | 37 / 42 | 88.10 | 39 / 42 | 92.86 |
| 9 | 20 / 42 | 47.62 | 21 / 42 | 50.00 |
| 10 | 26 / 42 | 61.90 | 27 / 42 | 64.29 |
| 11 | 38 / 42 | 90.48 | 38 / 42 | 90.48 |
| 12 | 42 / 42 | 100.00 | 42 / 42 | 100.00 |
| 13 | 34 / 41 | 82.93 | 34 / 41 | 82.93 |
| 14 | 37 / 42 | 88.10 | 37 / 42 | 88.10 |
| *15* | *Not included in the analysis* | | | |
| 16 | 33 / 42 | 78.57 | 33 / 42 | 78.57 |
| 17 | 29 / 42 | 69.05 | 30 / 42 | 71.43 |
| 18 | 42 / 42 | 100.00 | 42 / 42 | 100.00 |
| 19 | 35 / 42 | 83.33 | 35 / 42 | 83.33 |
| 20 | 38 / 42 | 90.48 | 39 / 42 | 92.86 |
| 21 | 30 / 42 | 71.43 | 30 / 42 | 71.43 |
| 22 | 17 / 42 | 40.48 | 18 / 42 | 42.86 |
| 23 | 24 / 42 | 57.14 | 24 / 42 | 57.14 |
| 24 | 24 / 42 | 57.14 | 25 / 42 | 59.52 |
| 25 | 37 / 42 | 88.10 | 38 / 42 | 90.48 |
| 26 | 42 / 42 | 100.00 | 42 / 42 | 100.00 |
| 27 | 40 / 42 | 95.24 | 40 / 42 | 95.24 |
| *28* | *Not included in the analysis* | | | |
| 29 | 36 / 42 | 85.71 | 38 / 42 | 90.48 |
| 30 | 39 / 42 | 92.86 | 39 / 42 | 92.86 |
| 31 | 18 / 42 | 42.86 | 19 / 42 | 45.24 |
| 32 | 35 / 42 | 83.33 | 35 / 42 | 83.33 |
| 33 | 32 / 42 | 76.19 | 34 / 42 | 80.95 |
| 34 | 35 / 42 | 83.33 | 35 / 42 | 83.33 |
| 35 | 38 / 42 | 90.48 | 39 / 42 | 92.86 |
| 36 | 42 / 42 | 100.00 | 42 / 42 | 100.00 |
| 37 | 16 / 42 | 38.10 | 21 / 42 | 50.00 |
| 38 | 25 / 42 | 59.52 | 29 / 42 | 69.05 |
| 39 | 40 / 42 | 95.24 | 40 / 42 | 95.24 |
| 40 | 34 / 42 | 80.95 | 34 / 42 | 80.95 |
| 41 | 38 / 42 | 90.48 | 38 / 42 | 90.48 |
| 42 | 32 / 42 | 76.19 | 34 / 42 | 80.95 |
| 43 | 21 / 42 | 50.00 | 23 / 42 | 54.76 |
| 44 | 38 / 42 | 90.48 | 39 / 42 | 92.86 |
| 45 | 39 / 42 | 92.86 | 40 / 42 | 95.24 |
| 46 | 42 / 42 | 100.00 | 42 / 42 | 100.00 |
| 47 | 41 / 42 | 97.62 | 41 / 42 | 97.62 |
| 48 | 21 / 42 | 50.00 | 22 / 42 | 52.38 |
| 49 | 25 / 39 | 64.10 | 26 / 39 | 66.67 |
| 50 | 30 / 30 | 100.00 | 30 / 30 | 100.00 |
| 51 | 16 / 42 | 38.10 | 17 / 42 | 40.48 |
| 52 | 37 / 42 | 88.10 | 37 / 42 | 88.10 |
| 53 | 24 / 42 | 57.14 | 24 / 42 | 57.14 |
| 54 | 40 / 42 | 95.24 | 40 / 42 | 95.24 |
| 55 | 34 / 42 | 80.95 | 35 / 42 | 83.33 |
| 56 | 20 / 34 | 58.82 | 20 / 34 | 58.82 |
| 57 | 27 / 42 | 64.29 | 27 / 42 | 64.29 |
| *58* | *Not included in the analysis* | | | |
| 59 | 32 / 42 | 76.19 | 35 / 42 | 83.33 |
| 60 | 28 / 42 | 66.67 | 29 / 42 | 69.05 |
| 61 | 37 / 42 | 88.10 | 38 / 42 | 90.48 |
| 62 | 27 / 42 | 64.29 | 27 / 42 | 64.29 |
| 63 | 36 / 42 | 85.71 | 36 / 42 | 85.71 |
| 64 | 18 / 42 | 42.86 | 20 / 42 | 47.62 |
| 65 | 38 / 42 | 90.48 | 38 / 42 | 90.48 |
| 66 | 25 / 42 | 59.52 | 26 / 42 | 61.90 |
| 67 | 32 / 42 | 76.19 | 32 / 42 | 76.19 |
