# Supplementary figures and images for: The variability of emotions, physical complaints, intention, and self-efficacy: an ecological momentary assessment study in older adults
Source: PeerJ. 2022 May 19;10:e13234. doi: 10.7717/peerj.13234 (PMC9124457; doi:10.7717/peerj.13234)

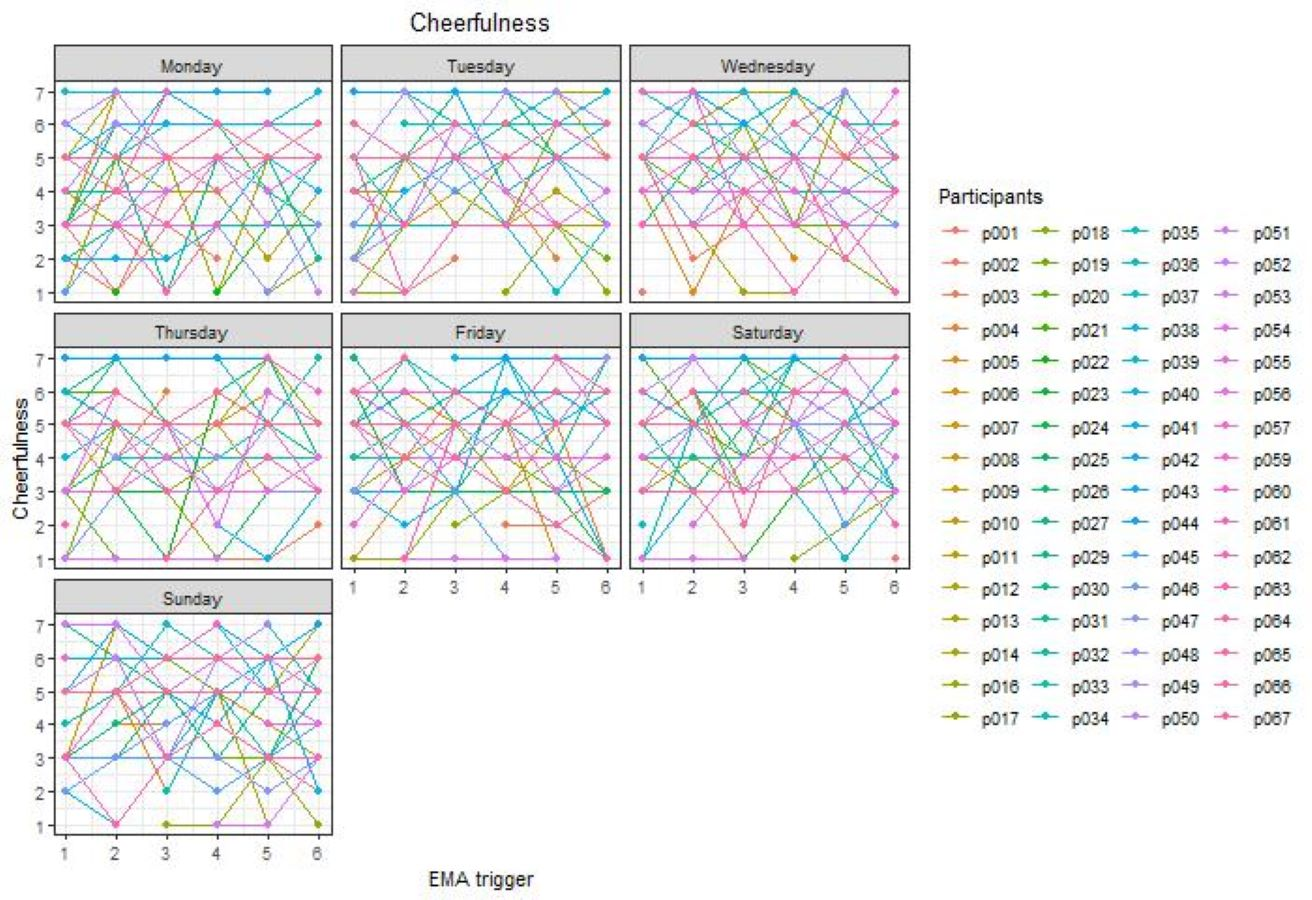

Supplement: Supplemental Information 7 [file peerj-10-13234-s007.png]

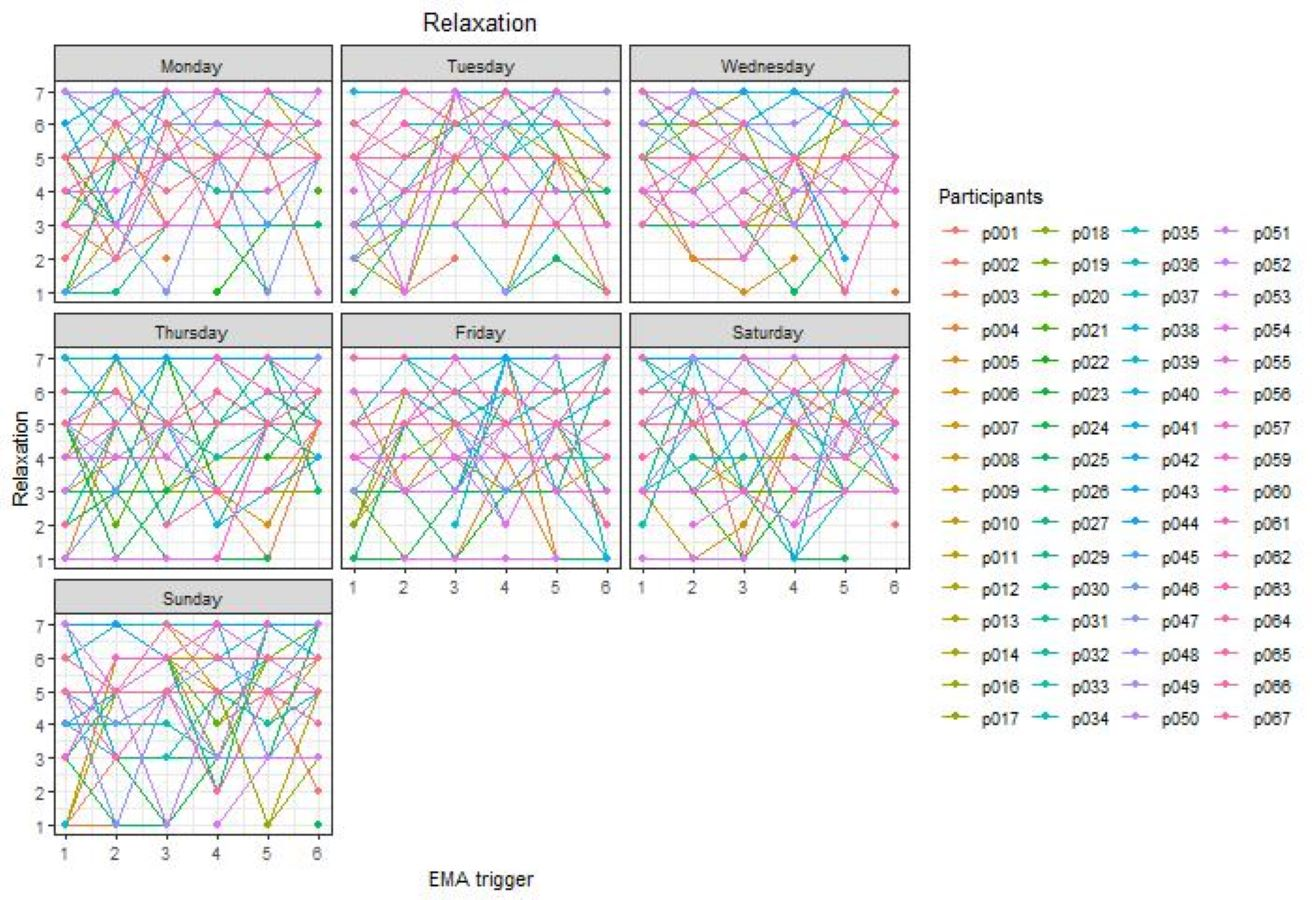

Supplement: Supplemental Information 8 [file peerj-10-13234-s008.png]

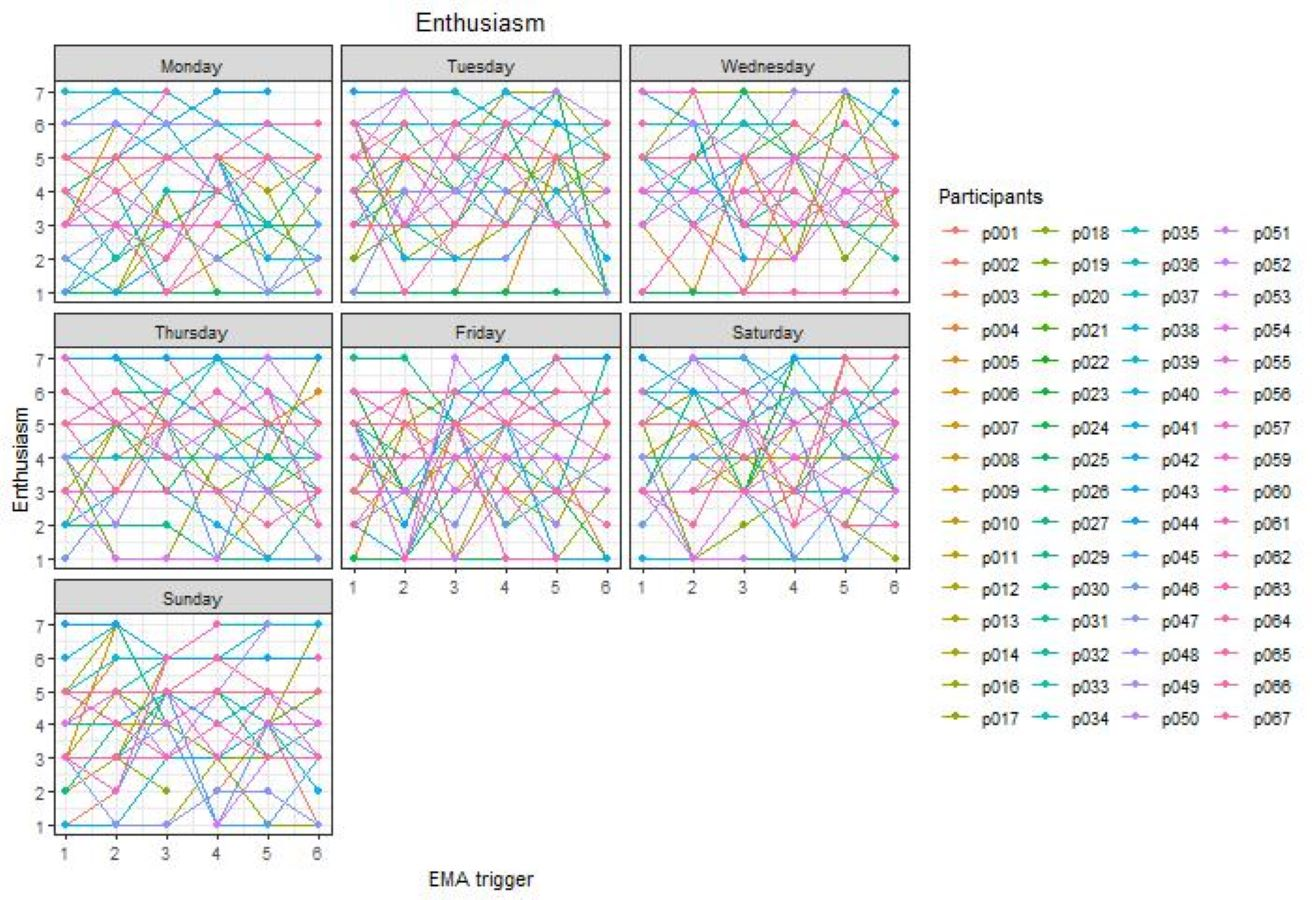

Supplement: Supplemental Information 9 [file peerj-10-13234-s009.png]

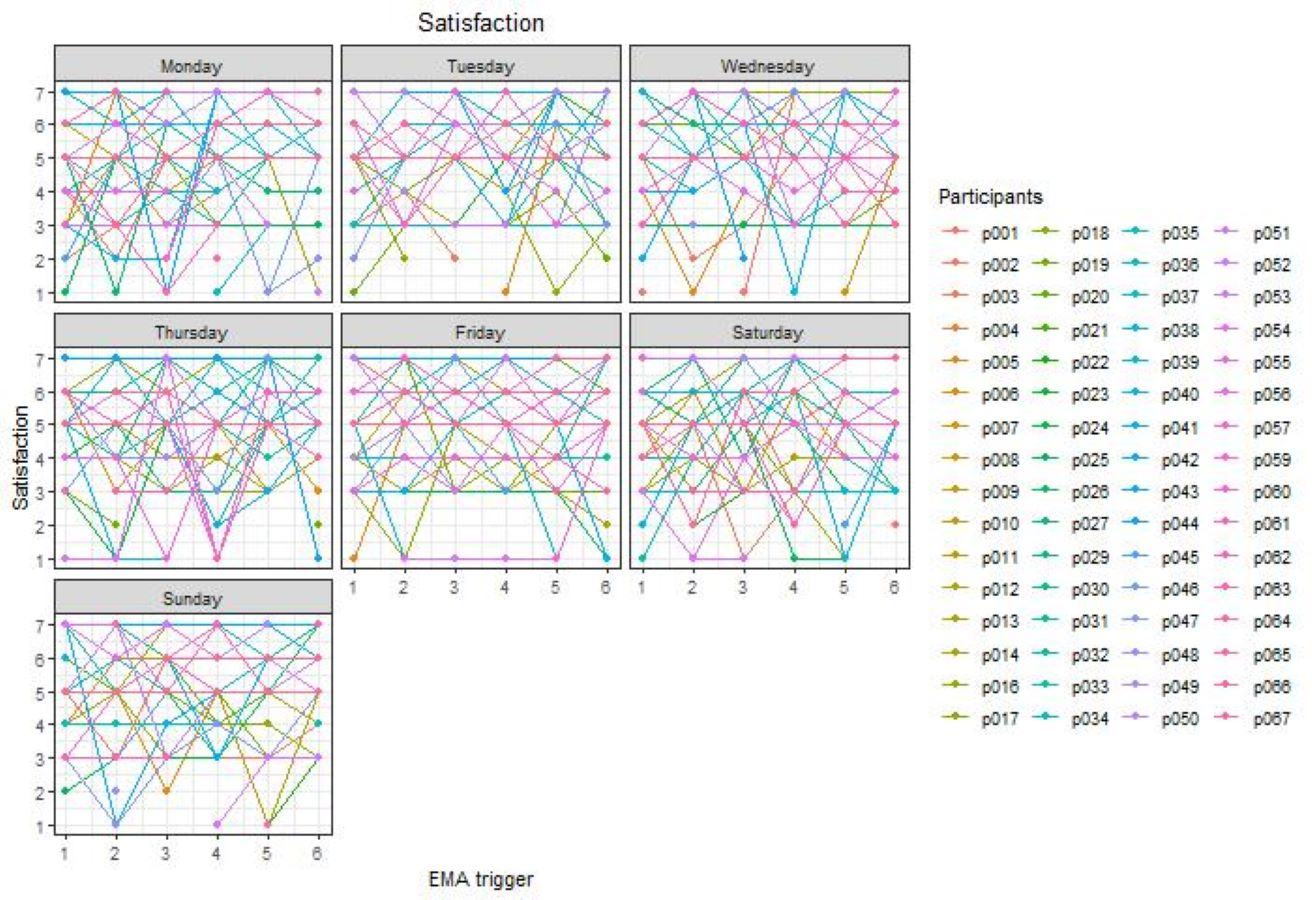

Supplement: Supplemental Information 10 [file peerj-10-13234-s010.png]

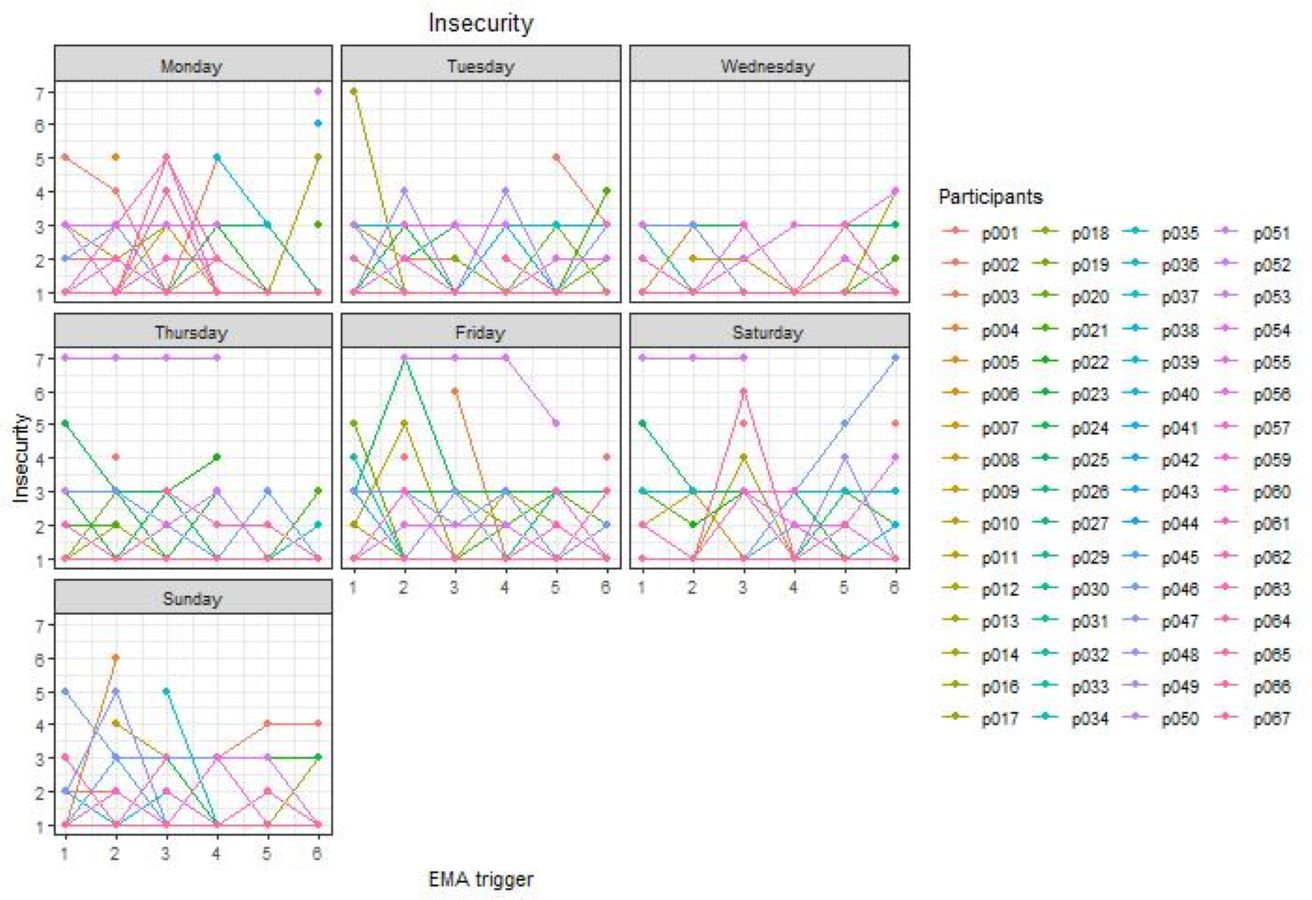

Supplement: Supplemental Information 11 [file peerj-10-13234-s011.png]

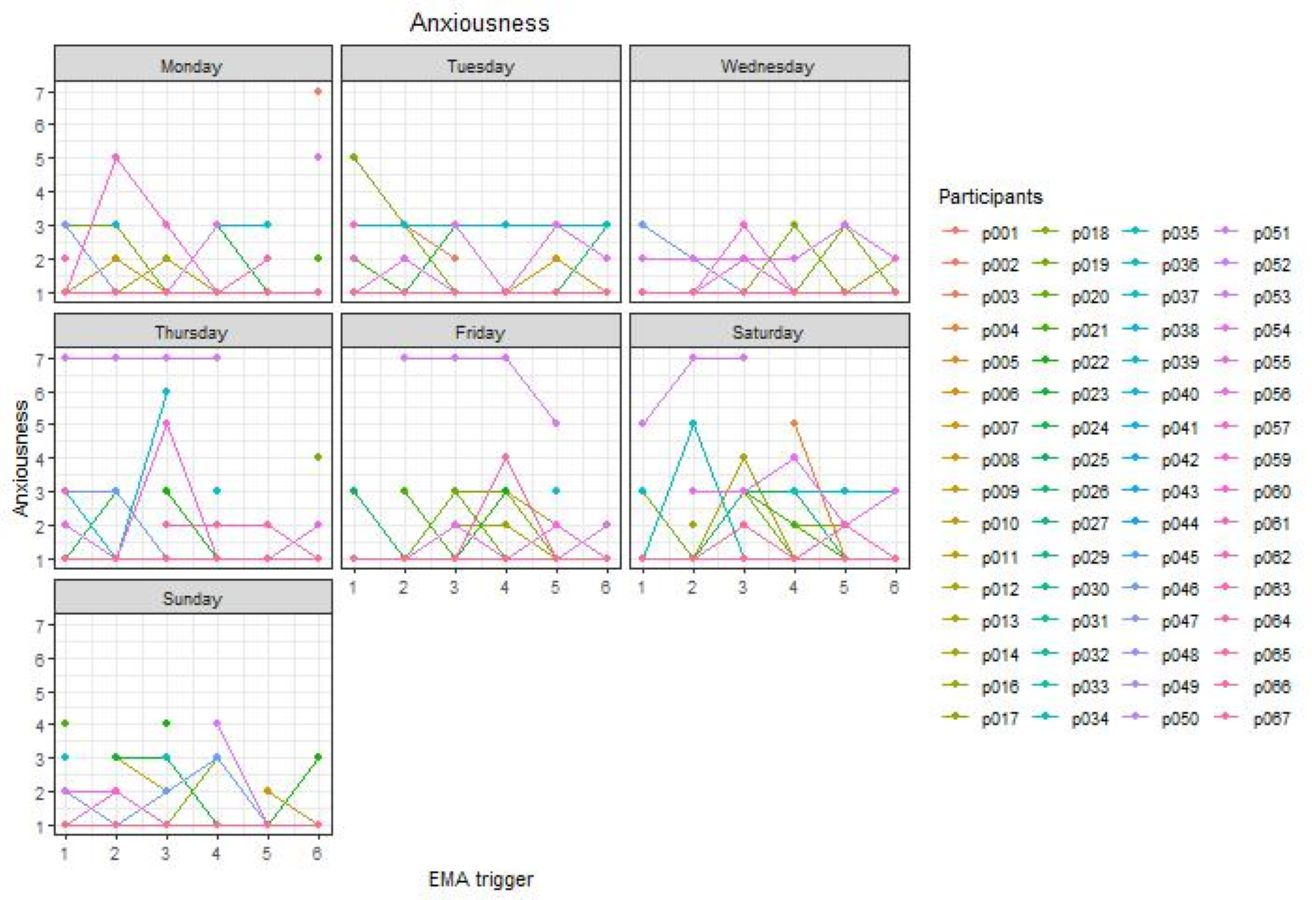

Supplement: Supplemental Information 12 [file peerj-10-13234-s012.png]

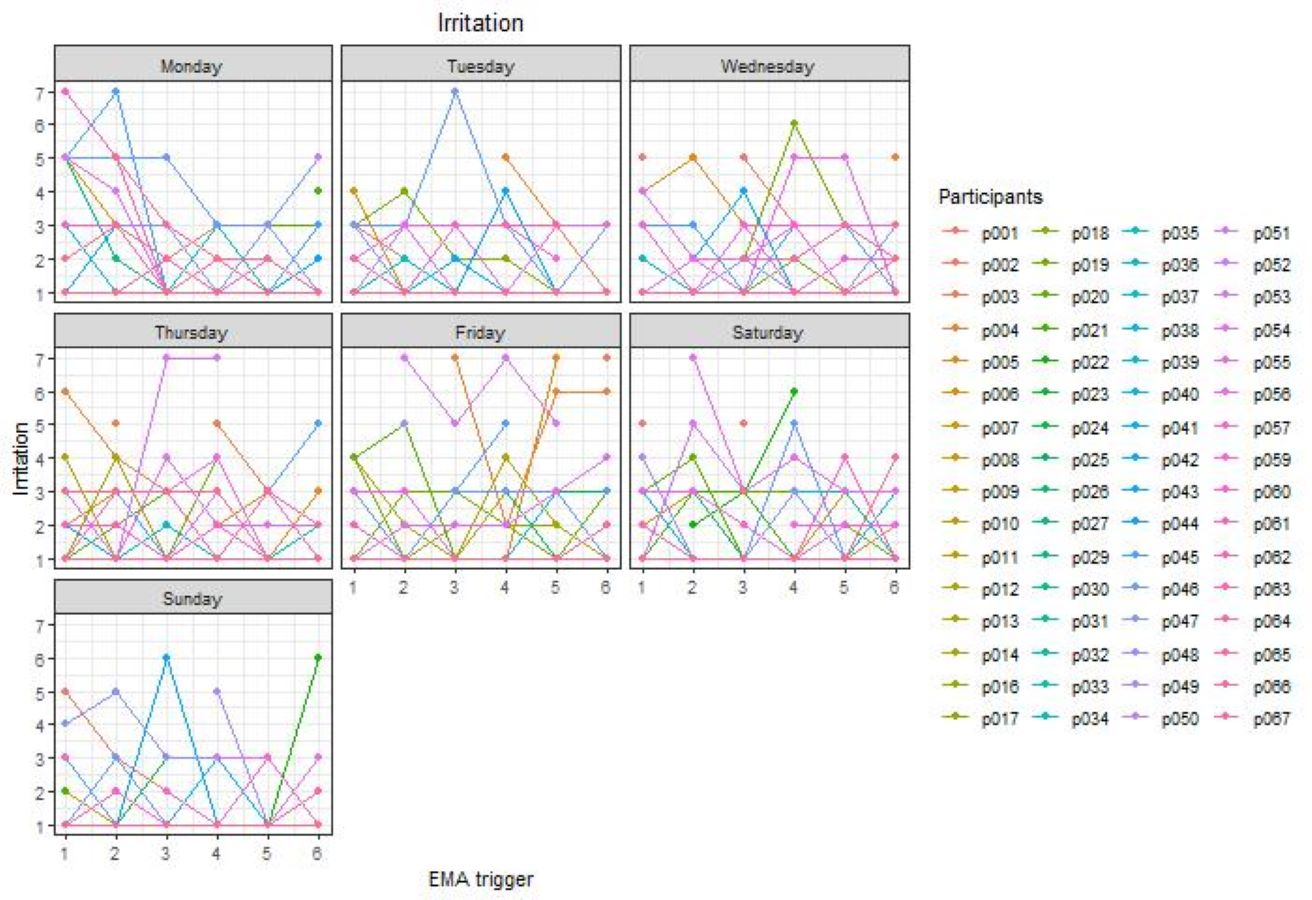

Supplement: Supplemental Information 13 [file peerj-10-13234-s013.png]

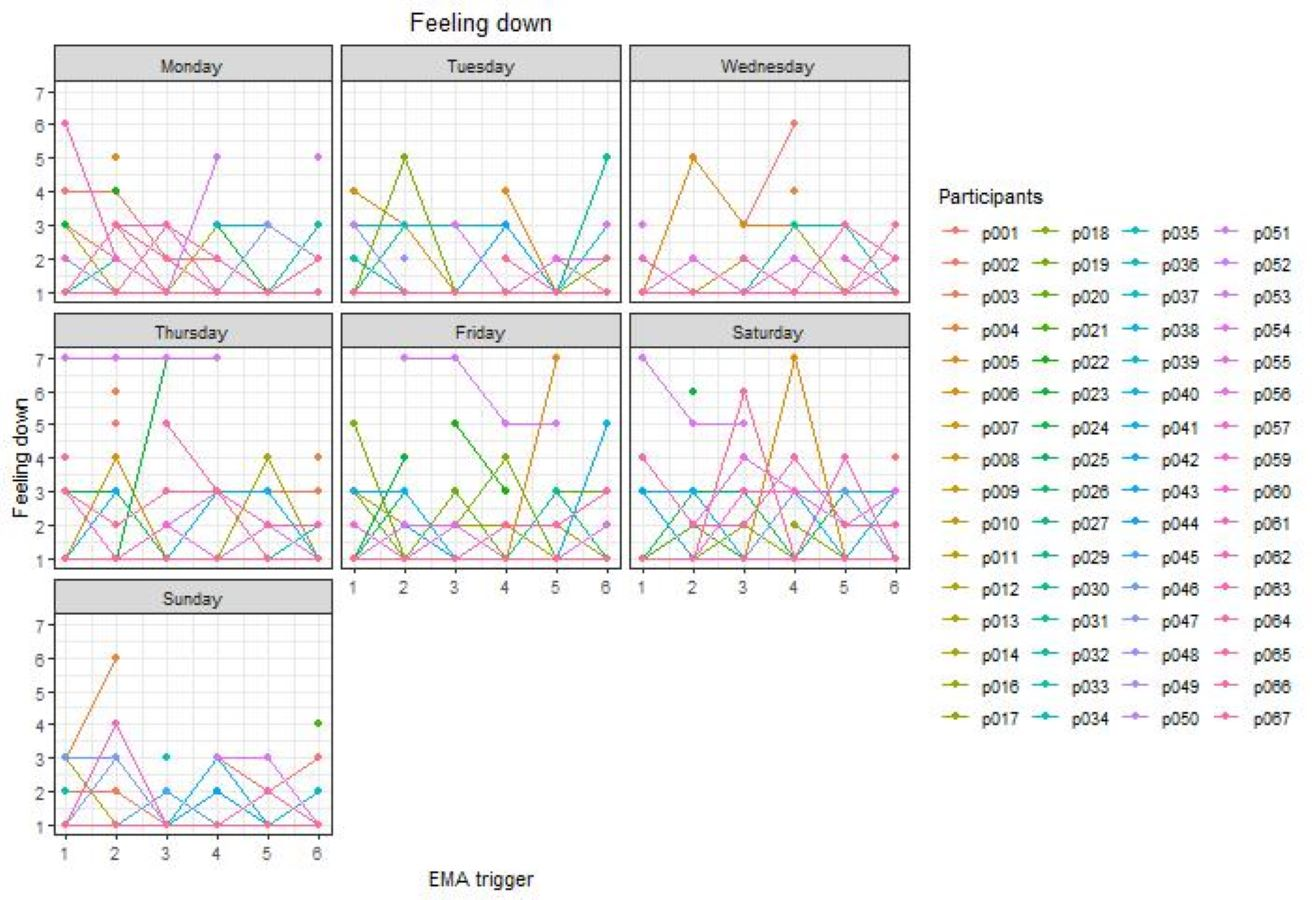

Supplement: Supplemental Information 14 [file peerj-10-13234-s014.png]

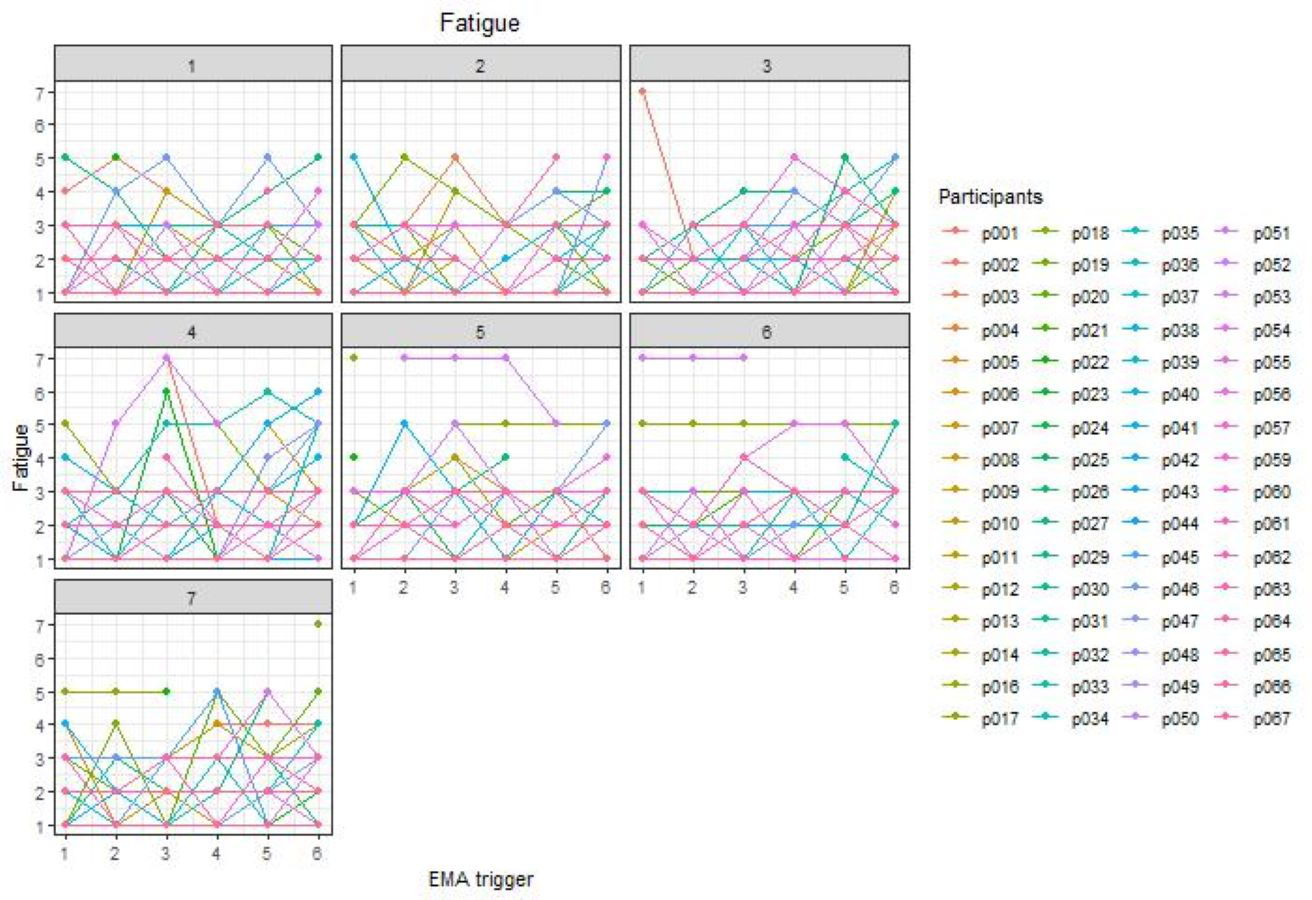

Supplement: Supplemental Information 15 [file peerj-10-13234-s015.png]

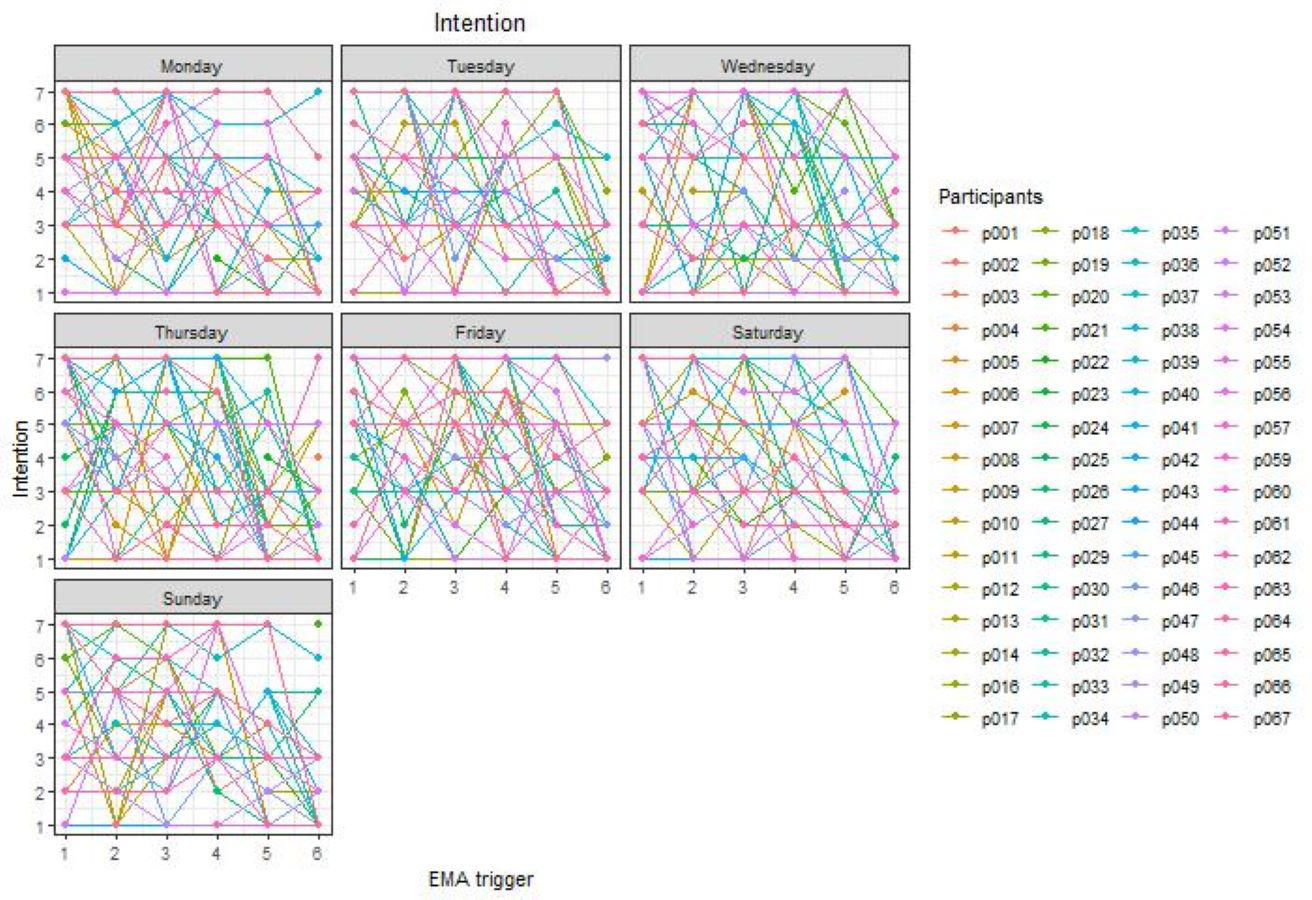

Supplement: Supplemental Information 16 [file peerj-10-13234-s016.png]

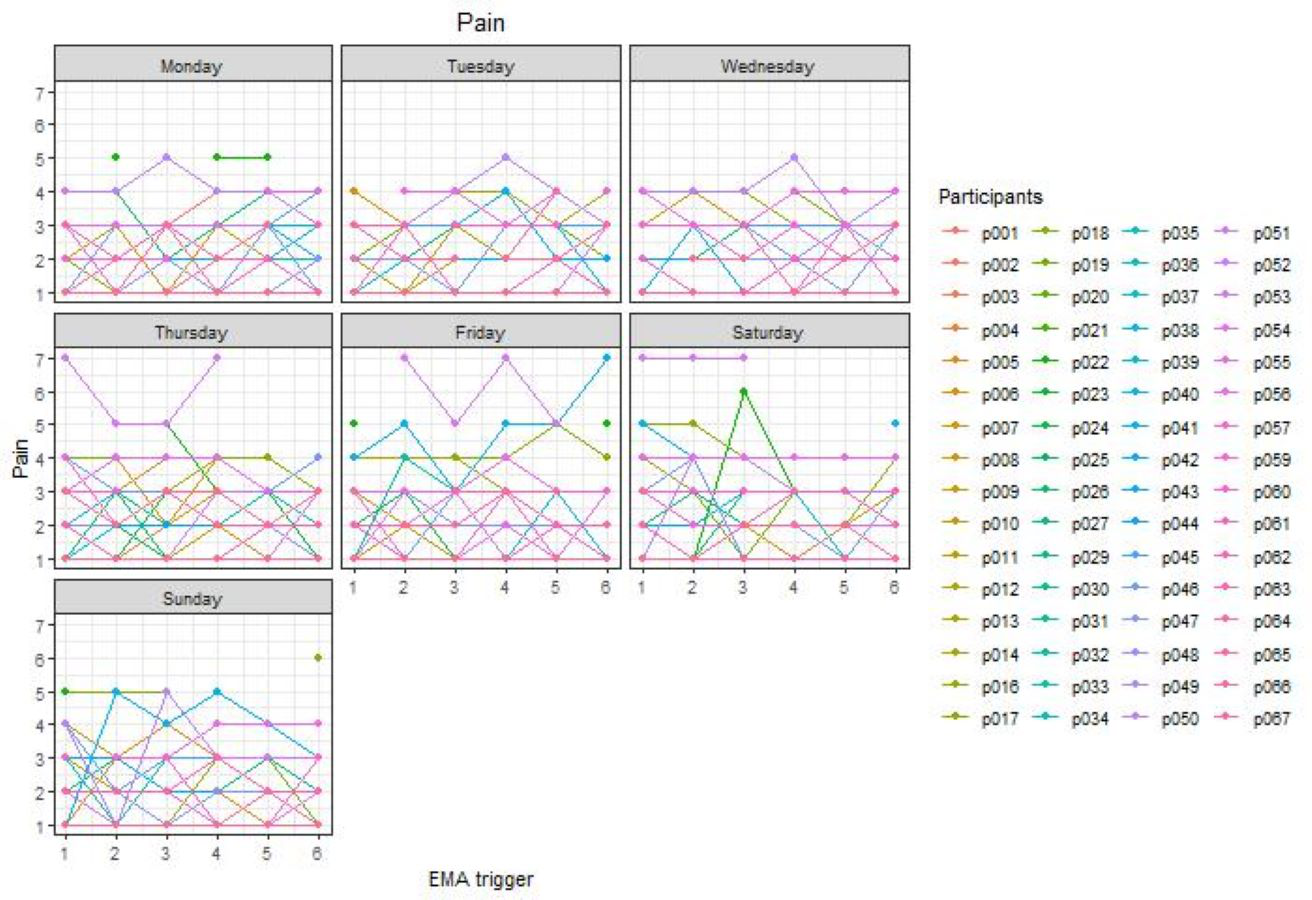

Supplement: Supplemental Information 17 [file peerj-10-13234-s017.png]

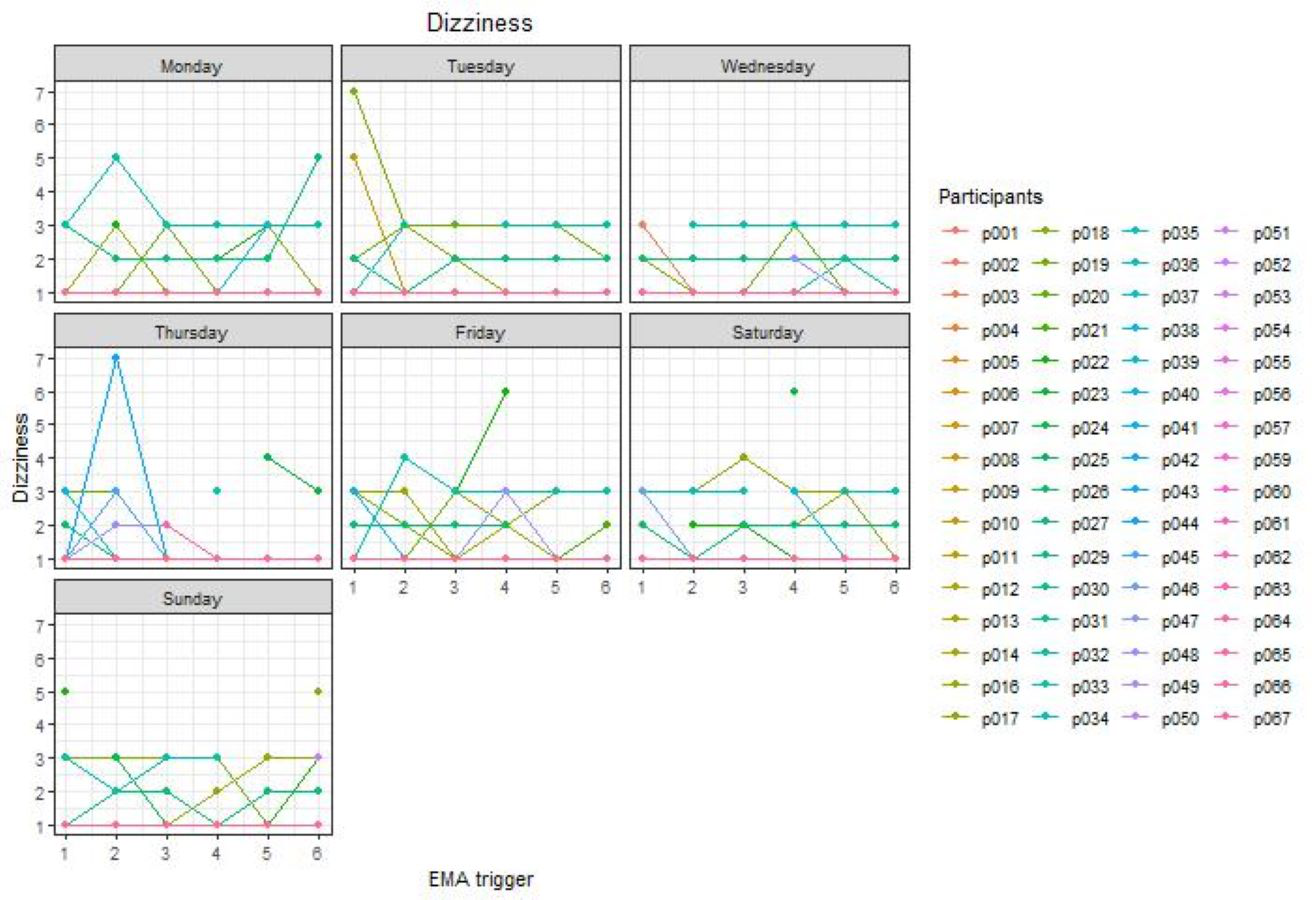

Supplement: Supplemental Information 18 [file peerj-10-13234-s018.png]

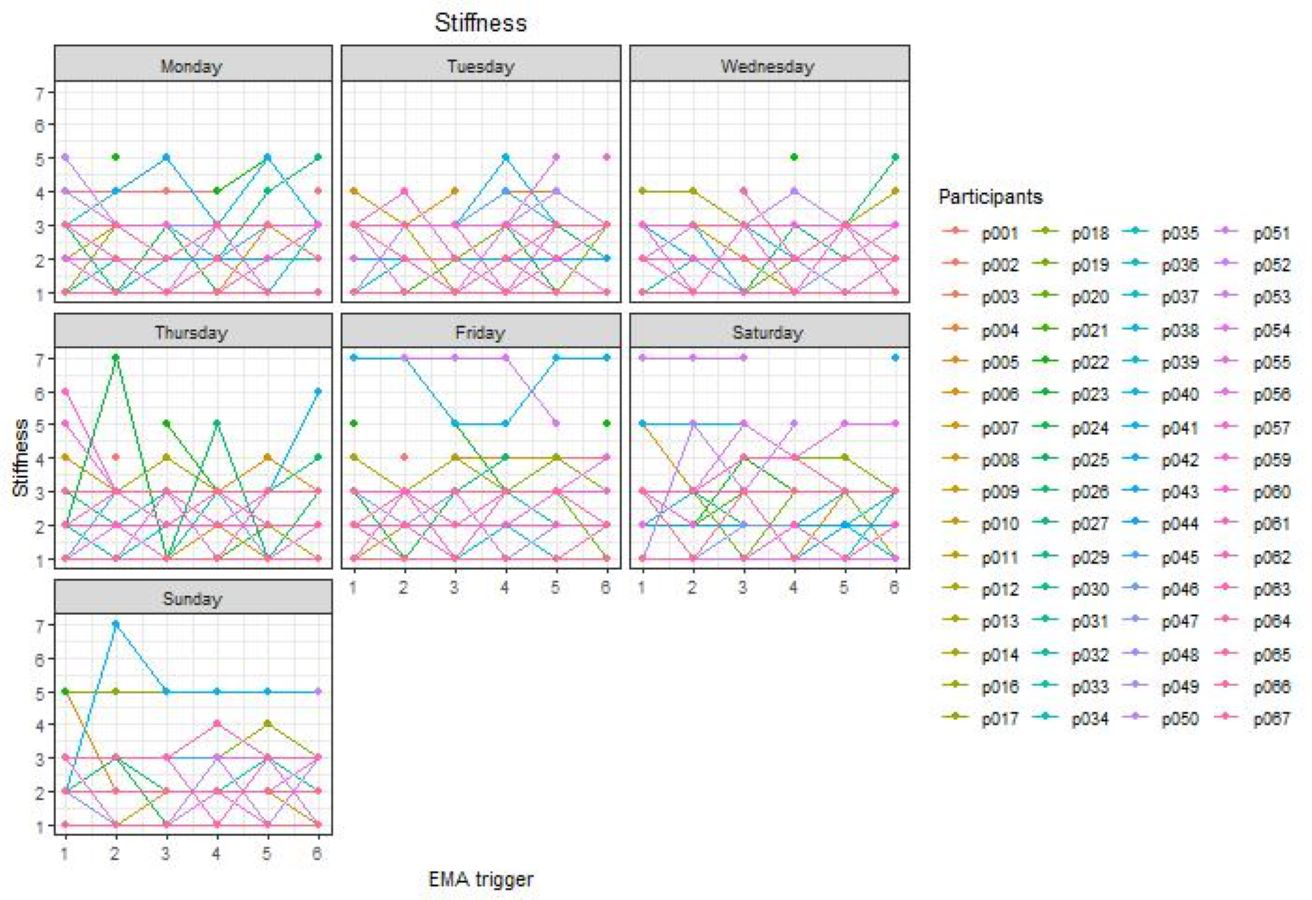

Supplement: Supplemental Information 19 [file peerj-10-13234-s019.png]

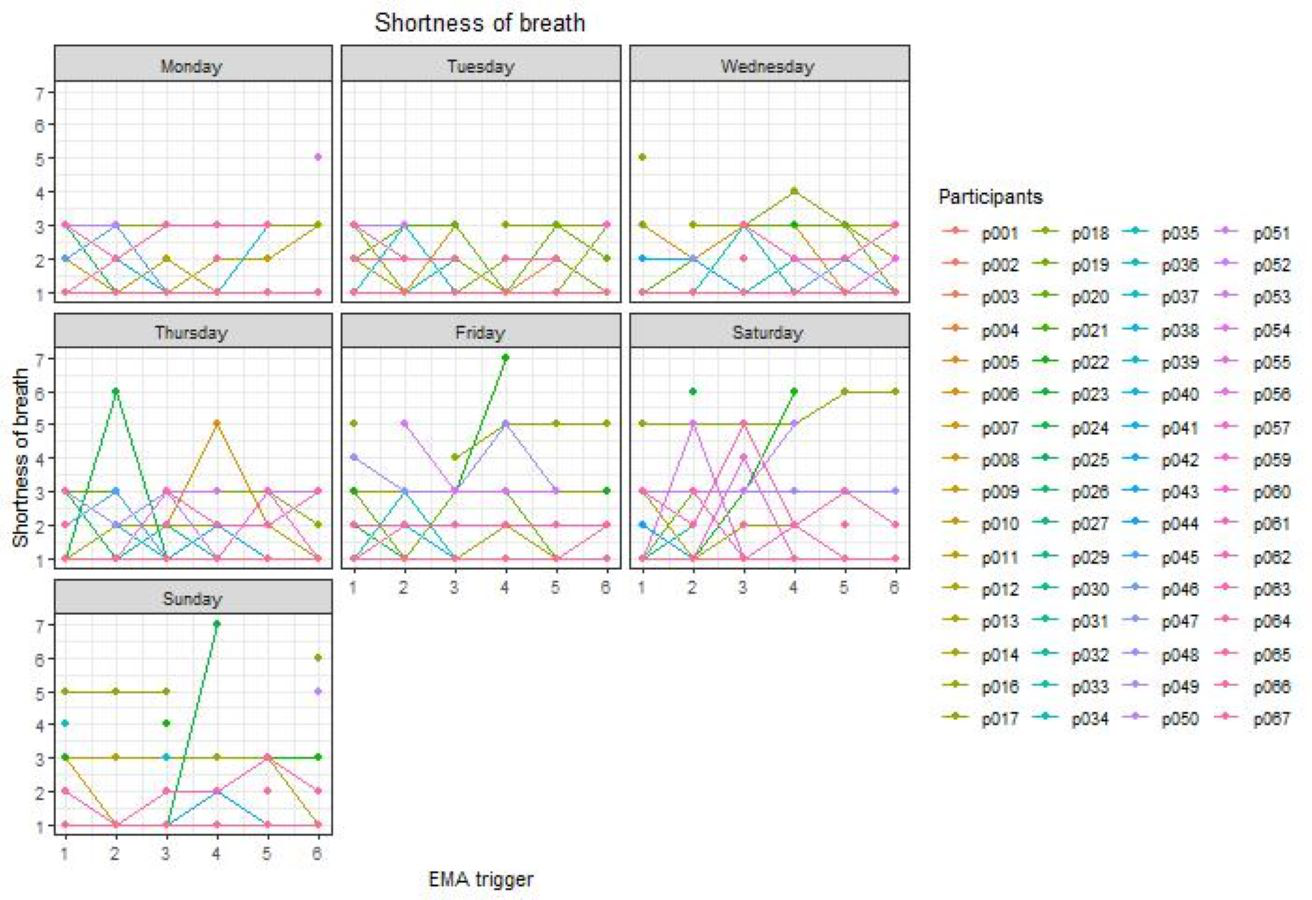

Supplement: Supplemental Information 20 [file peerj-10-13234-s020.png]

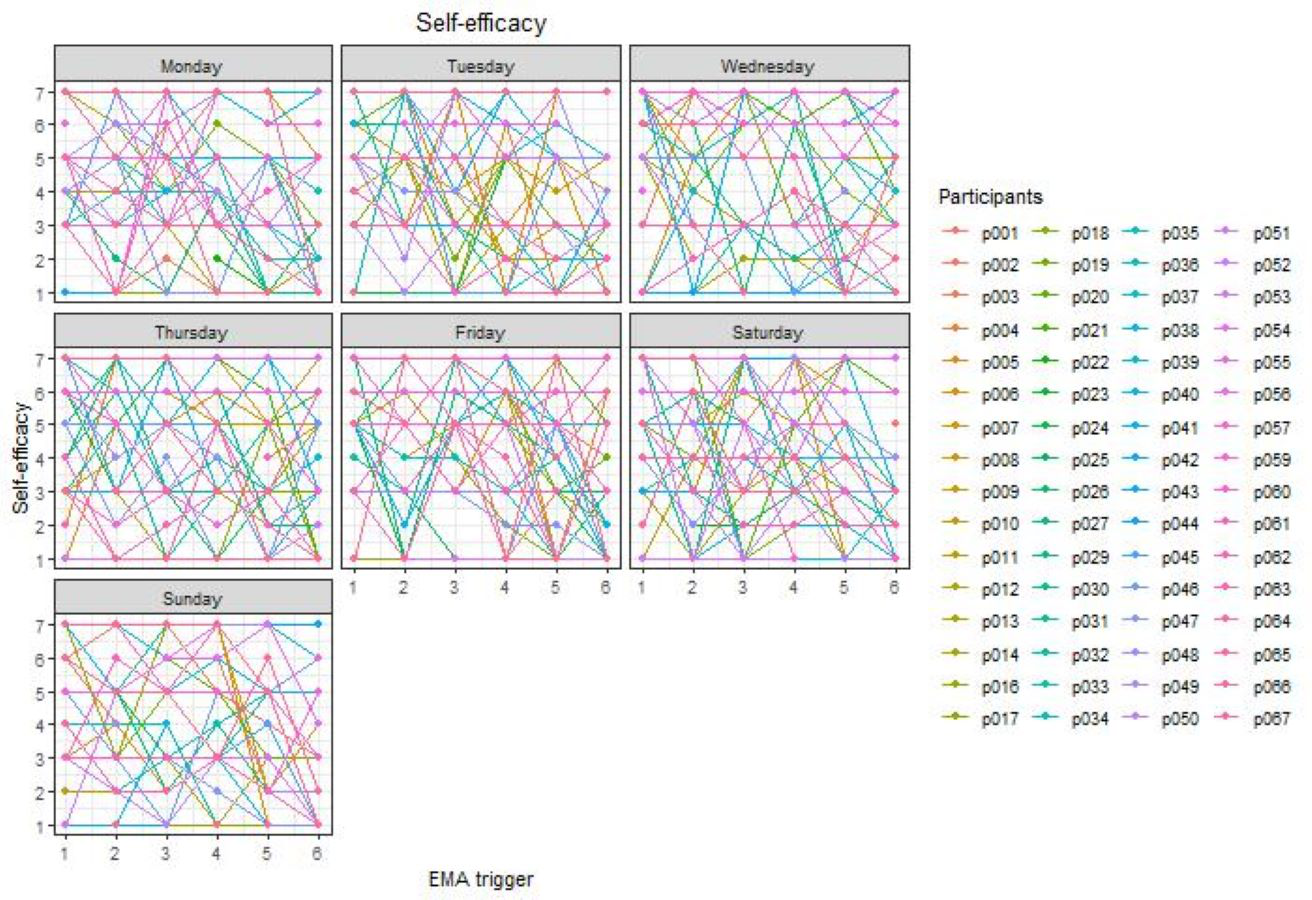

Supplement: Supplemental Information 21 [file peerj-10-13234-s021.png]
